# Immune signature-based risk stratification and prediction of immune checkpoint inhibitor’s efficacy for lung adenocarcinoma

**DOI:** 10.1007/s00262-020-02817-z

**Published:** 2021-01-02

**Authors:** Ming Yi, Anping Li, Linghui Zhou, Qian Chu, Suxia Luo, Kongming Wu

**Affiliations:** 1 Department of Oncology, Tongji Hospital of Tongji Medical College, Huazhong University of Science and Technology, Wuhan, 430030 China; 2 Department of Medical Oncology, The Affiliated Cancer Hospital of Zhengzhou University and Henan Cancer Hospital, Zhengzhou, 450008 China; 3 Bone Marrow Transplantation Center, The First Affiliated Hospital, Zhejiang University School of Medicine, No. 79 Qingchun Road, Hangzhou, 310003 China

**Keywords:** Lung adenocarcinoma, Immunotherapy, The tumor immune microenvironment, Immune checkpoint inhibitor, Tumor mutation burden, Prognostic model

## Abstract

**Background:**

Lung adenocarcinoma (LUAD) is a common pulmonary malignant disease with a poor prognosis. There were limited studies investigating the influences of the tumor immune microenvironment on LUAD patients’ survival and response to immune checkpoint inhibitors (ICIs).

**Methods:**

Based on TCGA-LUAD dataset, we constructed a prognostic immune signature and validated its predictive capability in the internal as well as total datasets. Then, we explored the differences of tumor-infiltrating lymphocytes, tumor mutation burden, and patients’ response to ICI treatment between the high-risk score group and low-risk score group.

**Results:**

This immune signature consisted of 17 immune-related genes, which was an independent prognostic factor for LUAD patients. In the low-risk score group, patients had better overall survival. Although the differences were non-significant, patients with low-risk scores had more tumor-infiltrating follicular helper T cells and fewer macrophages (M0), which were closely related to clinical outcomes. Additionally, the total TMB was markedly decreased in the low-risk score group. Using immunophenoscore as a surrogate of ICI response, we found that patients with low-risk scores had significantly higher immunophenoscore.

**Conclusion:**

The 17-immune-related genes signature may have prognostic and predictive relevance with ICI therapy but needs prospective validation.

## Background

In the United States, lung cancer is the first leading cause of cancer-related deaths in 2019 [1]. According to histology, lung cancer could be classified into small cell lung cancer (15% of all cases) and non-small cell lung cancer (NSCLC) (85% of all patients). NSCLC is further divided into three subtypes: adenocarcinoma, squamous carcinoma, and large cell carcinoma. Among these three subtypes, lung adenocarcinoma (LUAD) is most common and accounts for approximately 40% of lung cancer [2]. In the past decades, due to the advances of cancer genomics, a group of gene alterations is identified as driver gene mutations for LUAD, such as mutations in epidermal growth factor receptor (*EGFR*), *c-MET*, *KRAS*, anaplastic lymphoma kinase (*ALK*) [3–7]. Then, multiple agents targeting driver gene mutations have been developed. Unfortunately, after receiving targeting treatments, such as EGFR-tyrosine kinase inhibitors, most patients eventually become resistant to targeting therapy, partly because of secondary mutations in tumors [5].

Cancer immunology and immunotherapy provide a novel perspective for cancer therapeutics [8]. LUAD tends to have a high tumor mutation burden (TMB) and strong immunogenicity [9]. Therefore, LUAD is an ideal indication of immunotherapy [9]. In the clinic, immune checkpoint inhibitors (ICIs) targeting programmed cell death 1 (PD-1) and programmed cell death-ligand 1 (PD-L1) exhibit a potent and durable anti-tumor activity in LUAD patients [10]. However, the overall response rate of ICI is relatively low, and only a subset of LUAD patients could benefit from ICI treatment [11]. Up to now, a series of biomarkers have been verified to herald the efficacy of ICI treatment including TMB, PD-L1 expression level, neoantigens, gut microbiota, the status of immune cells [8]. Generally, most biomarkers reflect the status of the tumor immune microenvironment in a certain aspect. Recently, some computer algorithms, such as TIMER and CIBERSORT, make it feasible to estimate the immune profiling of cancer by transcriptome sequencing files [12, 13]. Constructing a comprehensive immune profiling-based model would be meaningful to predict the efficacy of ICI [14].

Actually, the immune landscape of cancer heralds the effect of immunotherapy and closely relates to patients’ prognosis [15]. Multiple previous studies indicated that some immune-related genes (IRGs) are prognostic biomarkers for colorectal cancer, ovarian cancer, and hepatocellular carcinoma [16–18]. There are few studies investigating the predictive value of IRGs and immune profiling in LUAD. In this study, we constructed an immune signature based on IRGs and explored the relationship between this immune signature and LUAD patients’ clinic-pathologic features as well as clinical outcomes. Additionally, we mapped the immune landscape, analyzed TMB, and predicted the response to ICI treatment in patients with different risk scores.

## Materials and methods

### Data acquiring and cleaning

LUAD patients’ transcriptome sequencing data and clinical information were download from UCSC Xena the Cancer Genome Atlas (TCGA) LUAD cohort (https://xenabrowser.net/). The mutation profiling was acquired from TCGA data portal (https://portal.gdc.cancer.gov/) by R software (version: 4.0.0) with package TCGAbiolinks [19]. Data cleaning was conducted by R software. The list of IRGs was downloaded from ImmPort database (https://immport.niaid.nih.gov) [20].

### Differentially expressed genes

To screen out the IRGs participating in the oncogenesis, we analyzed the differentially expressed genes between LUAD and corresponding normal tissues. By package edgeR, abnormally expressed genes were selected as previously described (adjusted *p* value < 0.05 and |log2 (fold change)|> 1) [21]. Differentially expressed IRGs were the intersection between IRGs and differentially expressed genes.

### Pathway and function enrichment analysis

To explore the biological significances of these differentially expressed IRGs, we performed pathway and function enrichment analysis with the online tool DAVID Bioinformatics Resources 6.8 (https://david.ncifcrf.gov/). Kyoto Encyclopedia of Genes and Genomes (KEGG) and gene ontology (GO) enrichment analyses were performed. Pathways and terms with false discovery rate < 0.05 were considered as significantly enriched objects. The visualization was performed by package ggplot2.

### Constructing an IRG-related immune signature for LUAD

The TCGA LUAD patients were randomly divided into a training set (2/3 for all patients) and a test set (1/3 for all patients). We used the training set to identify prognosis-related immune genes and constructed a prognostic risk model. Then, the predictive power and robustness of the model were validated by the test set and total patients. We first screened out prognosis-related immune genes by univariate Cox proportional hazard regression. To avoid overfitting, all genes with *p* value < 0.05 were involved in the subsequent least absolute shrinkage and selection operator (LASSO) analysis with package glmnet. After the filtration by LASSO model, the selected genes were used to construct the immune-related risk model by multivariate Cox proportional hazards model: risk score = level of gene *a* * coefficient *a* + level of gene *b* * coefficient *b* + level of gene *c* * coefficient *c* + …… + level of gene *n* * coefficient *n* [15]. In the model, the risk score reflected the prognosis of LUAD patients: the lower the score, the better the prognosis. Setting the median of risk score as the cutoff value, the patients were classified into a high-risk group and a low-risk group. The predictive power was calculated by Kaplan–Meier survival curves, and log-rank *p* value < 0.05 was regarded as statistically significant (with packages survival and survminer). To assess the predictive capability of this immune signature, time-dependent receiver operating characteristic curves were used (package survivalROC).

### Calculating the ratio of tumor-infiltrating immune cells

CIBERSORT could calculate the ratios of infiltrated immune cells from tissue transcriptional profiles by a deconvolution algorithm [13]. Based on TCGA LUAD transcriptional profiles and R script of CIBERSORT, we calculated the ratios of 22 types of tumor-infiltrating immune cells.

### TMB analysis

LUAD patients’ somatic variants data were analyzed and visualized by package maftools (the pipeline of MAF file: muse) [22]. Then, we calculated the TMB of each patient (mutations per million bases).

### Predicting the patients’ response to ICI

The Cancer Immunome Atlas (https://tcia.at/) analyzed the immune landscapes and antigenomes of 20 solid tumors. Tumor immunogenicity was quantitatively scored from 0 to 10, which was termed immunophenoscore (IPS) [23]. The IPS value was positively correlated to tumor immunogenicity. It has been verified that IPS could predict the patients’ response to ICI treatment [23]. We extracted data of IPS for the following analysis. Additionally, we compared the mRNA levels of immune checkpoints and their ligands in high-risk score group and low-risk score group.

### Statistical analysis

Differences among variables were tested by Student’s *t* test and Chi square test. Univariate and multivariate cox regression analyses were used to assess the influences of the immune signature and multiple clinic-pathological parameters on patients’ survival. Statistical analysis was performed by R software (4.0.0). The heat maps were plotted by package pheatmap. A two-sided *p* < 0.05 was regarded as statistically significant.

## Results

### The characteristics of patients

RNA-sequencing profiles and clinic-pathological data of 497 LUAD patients were downloaded from UCSC Xena TCGA-LUAD dataset. Patients were randomly divided into a training set (331 patients) and a test set (166 patients). For most clinic-pathological parameters, there was no significant difference between the training set and test set (Table 1).

**Table 1:** The clinical characteristics of LUAD patients

Variables	Group	Total set (*n* = 497)	Training set (*n* = 331)	Test set (*n* = 166)	*P* value
Vital status	Alive	317	206	111	0.36
	Dead	180	125	55	
Survival time		911.24	898.1	937.43	0.66
Clinical stage	I	267	187	80	0.17
	II	118	75	43	
	III	80	48	32	
	IV	25	17	8	
	NA	7	4	3	
T stage	T1-T2	433	288	145	0.64
	T3-T4	61	43	18	
	NA	3	0	3	
N stage	N0	321	223	98	0.11
	N1-N3	165	102	63	
	NA	11	6	5	
M stage	M0	331	226	105	0.97
	M1	24	17	7	
	NA	142	88	54	
Age	< 65	218	142	76	0.61
	≥ 65	279	189	90	

*NA* not available

Patients with not available data were excluded in statistical analysis

### Identifying differentially expressed genes

**Fig. 1:**
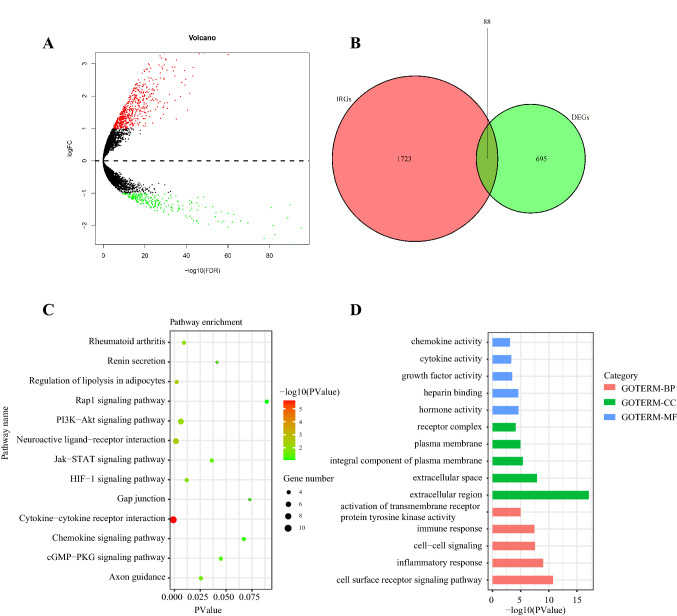
Identifying differentially expressed immune-related genes and enrichment analyses. **a** Volcano plot of differentially expressed genes between tumors and normal lung tissues. **b** Venn diagram of the intersection between differentially expressed genes and immune-related genes. **c** KEGG pathway enrichment analysis. **d** Gene ontology enrichment analysis. *BP* biology process; *CC* cellular component; *MF* molecular function

With the cutoff value adjusted *p* < 0.05 and |log2 (fold change)|> 1, 783 differentially expressed genes were screened out. Among them, 545 genes were upregulated, while 238 genes were downregulated in tumors (Fig. 1a). Based on the list of differentially expressed genes, 88 differentially expressed IRGs were selected (Fig. 1b). Pathway and function enrichment analyses were performed by the online annotation tool DAVID. The top five most enriched KEGG pathways were: cytokine–cytokine receptor interaction, neuroactive ligand–receptor interaction, regulation of lipolysis in adipocytes, PI3K-Akt signaling pathway, and rheumatoid arthritis (Fig. 1c). The top five most enriched GO- molecular function terms were: hormone activity, heparin binding, growth factor activity, cytokine activity, and chemokine activity (Fig. 1d).

### A 17-gene immune signature can predict the prognosis of LUAD patients

**Fig. 2:**
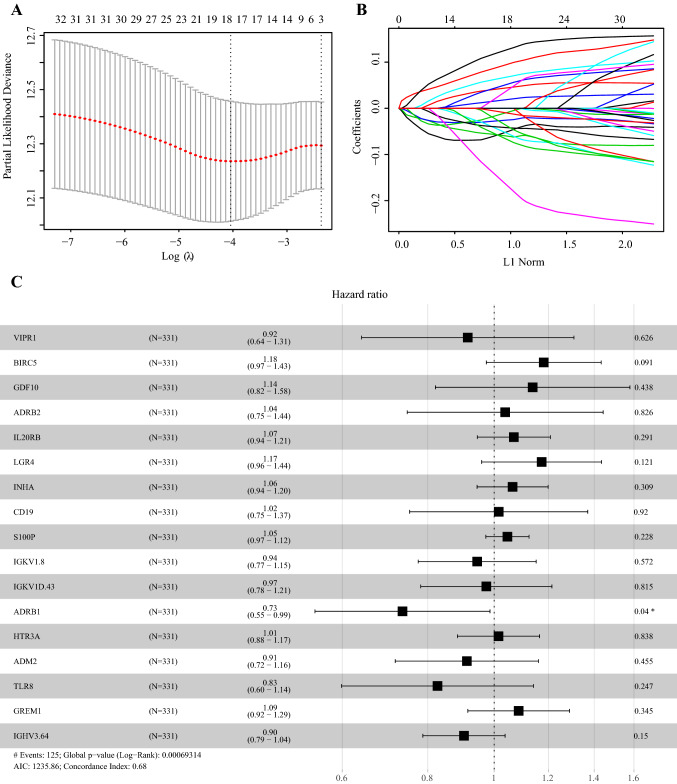
LASSO analysis and forest plot presenting the multivariable Cox model results of 17 immune-related genes. **a** cross-validation for tuning parameter selection in the LASSO model. **b** LASSO coefficient profiles of 32 prognostic immune-related genes. **c** Forest plot presenting the multivariable Cox model results

To explore the prognostic value of selected IRGs, we conducted a univariate Cox regression analysis. 32 genes were significantly associated to the overall survival (OS) in the training set. To avoid overfitting, we further conducted a LASSO analysis and 17 of 32 genes were predictors for patients’ prognosis (Fig. 2a–b). Then, based on the training set, we conducted a multivariate Cox proportional hazards regression analysis and established a predictive model: risk score = (VIPR1*-0.08876) + (BIRC5*0.16676) + (GDF10*0.12945) + (ADRB2*0.03706) + (IL20RB*0.06621) + (LGR4*0.15962) + (INHA*0.06204) + (CD19*0.01529) + (S100P*0.04467) + (IGKV1.8*-0.05711) + (IGKV1D.43*-0.02639) + (ADRB1*-0.30831) + (HTR3A*0.01446) + (ADM2*-0.09179) + (TLR8*-0.19053) + (GREM1*0.08239) + (IGHV3.64*-0.10157) (Fig. 2c). Seven genes were protective factors (*IGKV1D.43*, *IGKV1.8*, *VIPR1*, *ADM2*, *IGHV3.64*, *TLR8*, and *ADRB1*) while ten genes were risk factors (*BIRC5*, *LGR4*, *GDF10*, *GREM1*, *IL20RB*, *INHA*, *S100P*, *ADRB2*, *CD19*, and *HTR3A*).

**Fig. 3:**
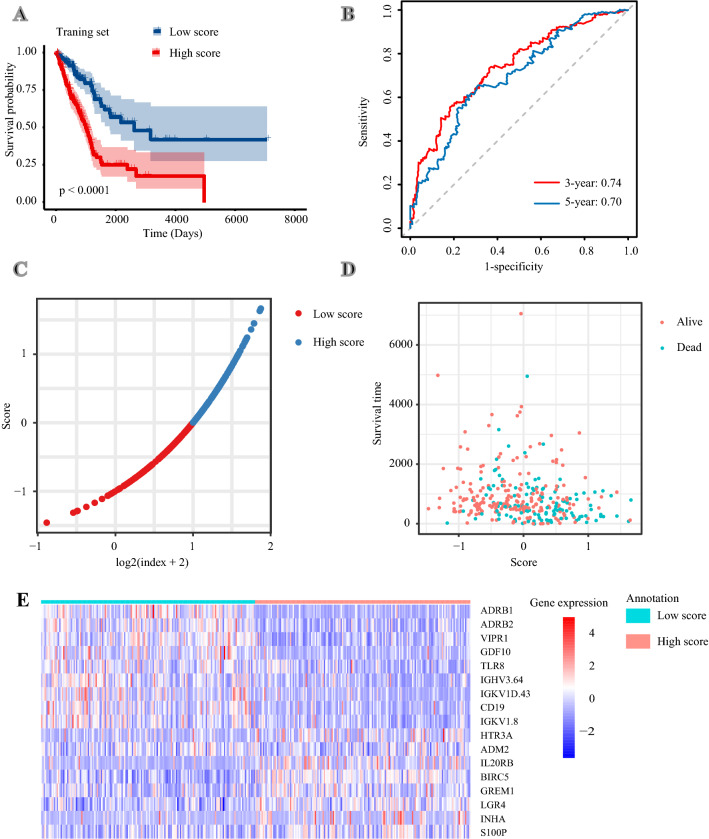
Constructing a 17-immune-related gene signature in the training set. **a** Kaplan–Meier curves of overall survival of LUAD patients in high- and low-risk score groups. **b** Time-dependent receiver operating characteristic curves. **c** The distribution of risk scores. **d** The relationship between risk scores and survival times. **e** The expression patterns of 17 immune-related genes in high- and low-risk score groups

According to the formula mentioned above, the risk score of each patient in the training set was calculated. Patients were classified into the high-risk group and low-risk group using the median risk score as the cutoff value. Patients in the high-risk group had a significantly poorer OS (*P* < 0.0001) (Fig. 3a). The areas under the curves (AUCs) of this immune signature were 0.74 for 3-year OS and 0.70 for 5-year OS (Fig. 3b). Besides, we ranked patients’ risk scores and overviewed their distributions (Fig. 3c). Patients’ risk scores and survival times were presented in the dot plot (Fig. 3d). The heat map exhibited the expression patterns of 17 IRGs in patients with different risk scores (Fig. 3e).

**Fig. 4:**
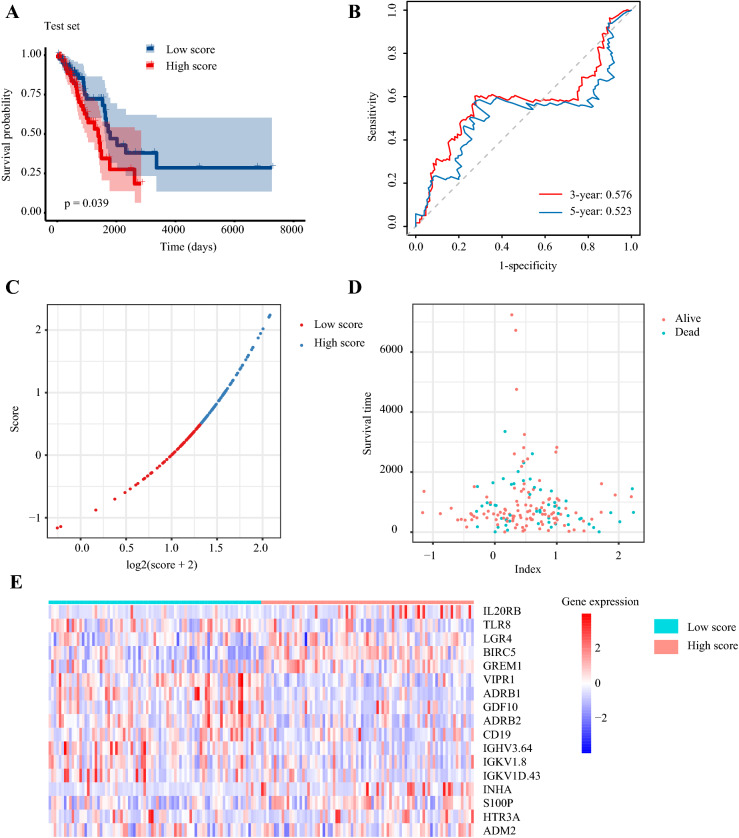
Validating the 17-immune-related gene signature in the test set. **a** Kaplan–Meier curves of overall survival of LUAD patients in high- and low-risk score groups. **b** Time-dependent receiver operating characteristic curves. **c** The distribution of risk scores. **d** The relationship between risk scores and survival times. **e** The expression patterns of 17 immune-related genes in high- and low-risk score groups

The predictive capability of this immune signature was further verified in the testing set. According to the predictive signature, each patient in the testing set was divided into the high-risk group and low-risk group as previously described. Survival analysis showed that patients with low-risk scores had longer OS (*p* = 0.039) (Fig. 4a). The AUC of 3-year OS was 0.58, and the AUC of 5-year OS was 0.52 (Fig. 4b). The distribution of risk score, patients’ survival status, and survival were presented by scatter plots (Fig. 4c–d). The expression of 17 selected genes was visualized by heat map (Fig. 4e).

**Fig. 5:**
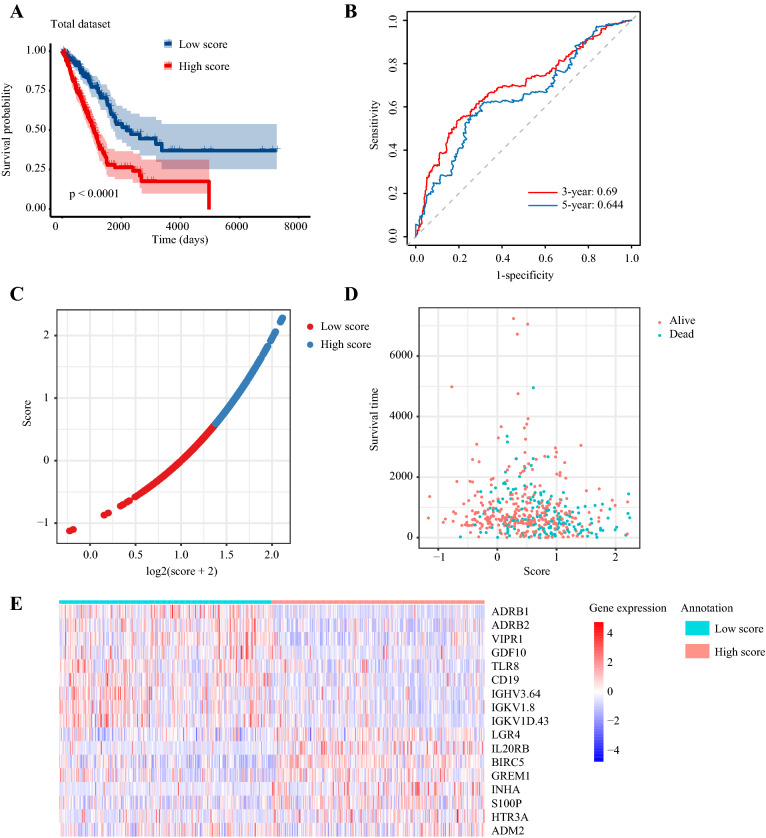
Validating the 17-immune-related gene signature in the total dataset. **a** Kaplan–Meier curves of overall survival of LUAD patients in high- and low-risk score groups. **b** Time-dependent receiver operating characteristic curves. **c** The distribution of risk scores. **d** The relationship between risk scores and survival times. **e** The expression patterns of 17 immune-related genes in high- and low-risk score groups

Besides, the robustness of the 17-IRG signature was assessed in the total set. The high-risk score was an unfavorable factor of LUAD patients (*p* < 0.0001) (Fig. 5a). The AUC of 3-year OS was 0.69, and the AUC of 5-year OS was 0.64 (Fig. 5b). The distribution of risk score, patients’ vital status, OS time, and 17 IRGs expression were also presented in Fig. 5c–e.

### The 17-gene immune signature is an independent prognostic factor for LUAD patients

We performed a univariate Cox regression analysis to assess the influences of patients’ clinic-pathological factors and immune signature risk score on patients’ OS in the total set (Table 2). The results indicated that bigger tumor size, lymph node metastasis, distant metastasis, advanced TNM stage, and high-risk score were unfavorable factors for OS. Then, we performed a multivariate Cox regression analysis. It was found that the risk score of the 17-IRG signature was an independent prognostic factor (HR = 2.2, 95% CI 1.51 ~ 3.2; *p* < 0.0001).

**Table 2:** Univariate and multi-variate Cox regression analysis

Variables	Univariate analysis		Multivariate analysis	
	HR (95%CI)	*P* value	HR (95%CI)	*P* value
Age (≥ 65 vs. < 65 years)	1.09 (0.809 ~ 1.47)	0.57		
T stage (T3-T4 vs. T1-T2)	2.28 (1.55 ~ 3.35)	< 0.0001	1.7 (1.06 ~ 2.8)	0.029
N stage (N1-N3 vs. T0)	2.64 (1.96 ~ 3.56)	< 0.0001	2.1 (1.44 ~ 3.2)	< 0.0001
M stage (M1 vs. M0)	2.13 (1.24 ~ 3.65)	0.01	1.4 (0.71 ~ 2.6)	0.34
TNM stage (stage III-IV vs. I-II)	2.63 (1.93 ~ 3.59)	< 0.0001	1.2 (0.73 ~ 2.0)	0.44
Risk score (high vs. low)	2.52 (1.85 ~ 3.43)	< 0.0001	2.2 (1.51 ~ 3.2)	< 0.0001

### The associations between 17-IRG signature and patients’ clinic-pathological parameters

**Fig. 6:**
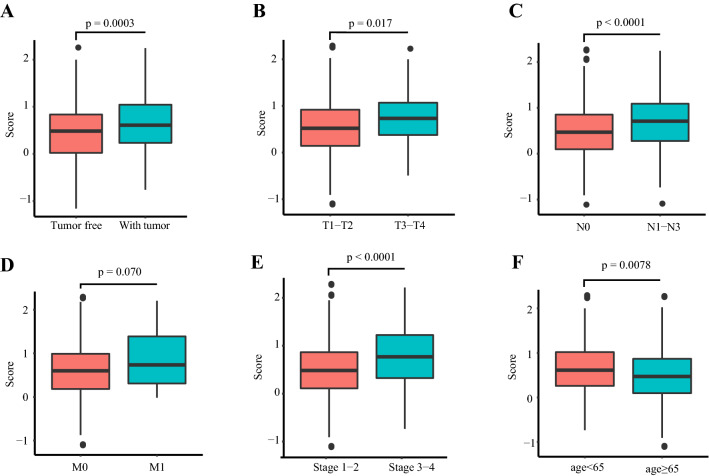
The relationships between the immune signature and patients’ clinic-pathologic parameters. **a** Tumor burden. **b** Tumor size. **c** Lymph node status. **d** Distant metastasis. **e** TNM stage. **f** Age at diagnosis

We analyzed the relationships between this 17-IRG signature and patients’ clinic-pathological parameters, including tumor burden, tumor size, lymph node status, distant metastasis, TNM stage, and age at diagnosis (Fig. 6a–f). The results showed that this 17-IRG risk score was significantly higher in patients with higher tumor burden, bigger tumor size, lymph node metastasis, advanced TNM stage, and age below 65. Besides, the risk score was higher in patients with distant metastasis, and the difference was on the verge of statistical significance (*p* = 0.070).

### The 17-IRG signature and tumor immune microenvironment

**Fig. 7:**
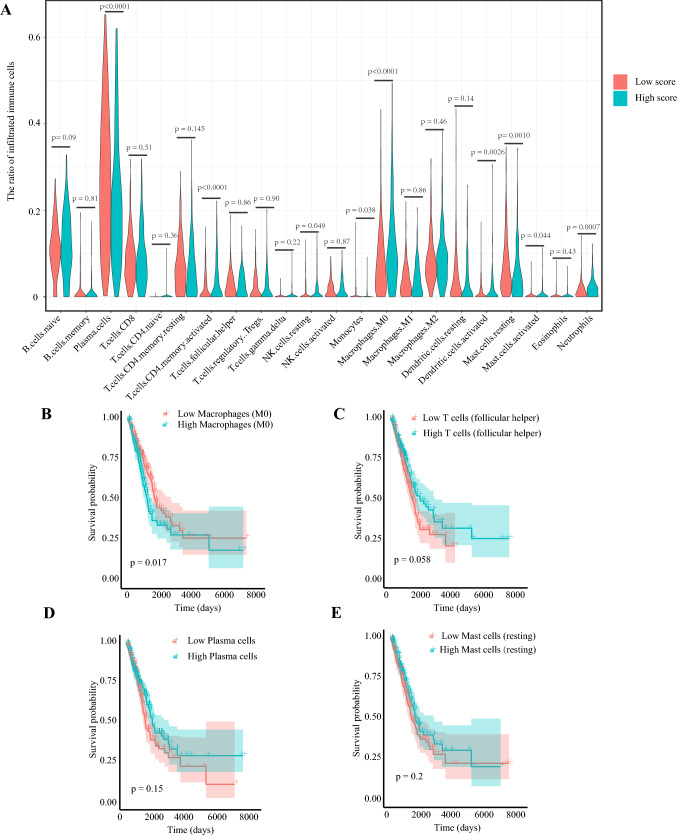
The relationships between tumor-infiltrating immune cells and risk scores, as well as patients’ overall survival. **a** The association between tumor-infiltrating immune cells and the immune risk signature. The associations between overall survival and (**b**) Macrophages (M0), (**c**) Follicular helper T cells, (**d**) Plasma cells, (**e**) Resting mast cells

Based on the CIBERSORT algorithm, we calculated the proportions of 22 types of immune cells in each LUAD sample. Then, we compared the differences in proportions of immune cells between the high-risk score group and low-risk score group. It was found that the ratios of plasma cell, monocyte, and resting mast cell were significantly higher in the low-risk score group. On the contrary, the proportions of activated memory CD4^+^ T cell, resting NK cell, macrophage (M0), activated dendritic cell, activated mast cell, and neutrophil were significantly higher in the high-risk score group (Fig. 7a). In 22 types of immune cells, high macrophage (M0) level was significantly related to poor OS while increased follicular helper T cell, plasma cell, and resting mast cell were related to better OS (on the verge of statistical significance, *p* = 0.058, 0.15, 0.20, respectively) (Fig. 7b–e).

### The immune signature and TMB

**Fig. 8:**
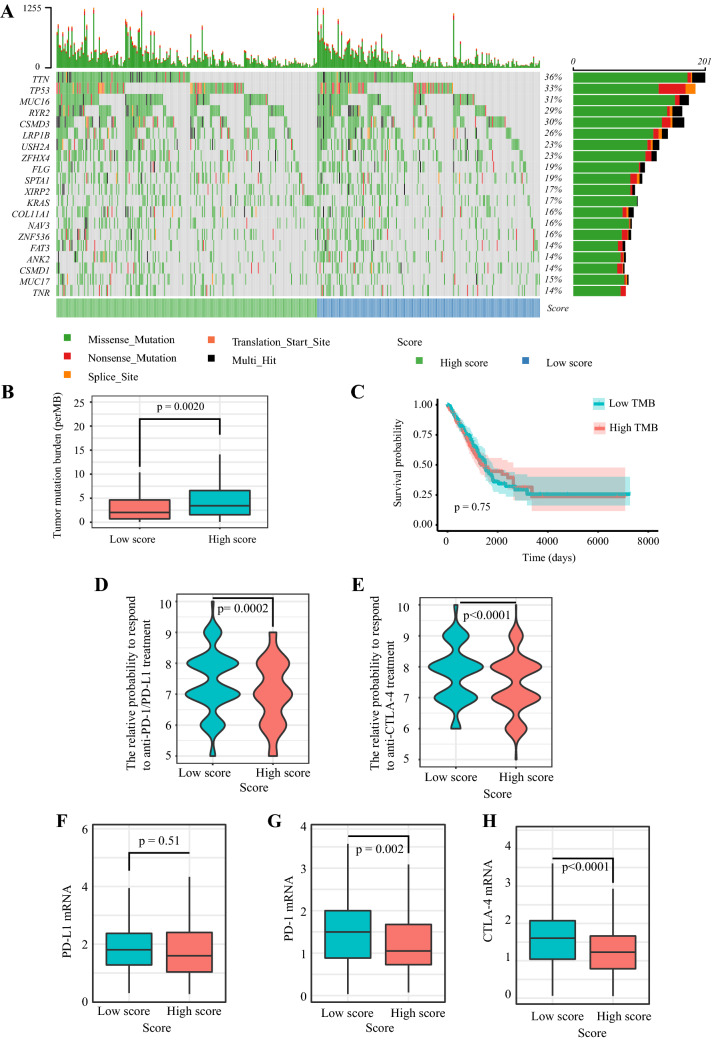
The mutation profile, TMB, relative probabilities to respond to immune checkpoint inhibitors, and the levels of immune checkpoint molecules in low-risk and high-risk groups. **a** Mutation profile of LUAD patients in low-risk and high-risk groups. **b** The difference of tumor mutation burden between low-risk and high-risk groups. **c** The association of tumor mutation burden and patients’ overall survival. **d**–**e** The relative probabilities to respond to anti-PD-1/PD-L1 and anti-CTLA-4 treatment in the low-risk score and high-risk score group. The expressions of (**f**) PD-L1, (**j**) PD-1, and (**h**) CTLA-4 in low-risk and high-risk groups

The mutation profiles of each LUAD patient were analyzed. For the total set, the top 20 most significantly mutated genes were *TTN*, *TP53*, *MUC16*, *RYR2*, *CSMD3*, *LRP1B*, *TNR*, *MUC17*, *CSMD1*, *ANK2*, *FAT3*, *ZNF536*, *NAV3*, *COL11A1*, *KRAS*, *XIRP2*, *SPTA1*, *FLG*, *ZFHX4*, *USH2A* (Fig. 8a). Subsequently, we calculated the TMB of each sample and found TMB was markedly higher in the high-risk score group (*p* = 0.0020) (Fig. 8b). However, we observed that TMB was not related to patients’ OS (*p* = 0.75) (Fig. 8c).

### The immune signature and patients’ response to ICI treatment

IPS is a machine learning-based scoring scheme, which could predict patients’ response to immune checkpoint inhibitor in silico [23]. Given that the information on ICI treatment was not available in TCGA LUAD dataset, we used two subtypes of IPS values (IPS-PD-1/PD-L1/PD-L2_pos and IPS-CTLA-4_pos) as the surrogates of the LUAD patients’ responses to anti-PD-1/PD-L1 and anti-CTLA-4 treatment. In this predictive model, the relative probabilities to respond to anti-PD-1/PD-L1 and anti-CTLA-4 treatment were higher in the low-risk score group (*p* = 0.0002 and *p* < 0.0001) (Fig. 8d–e). The results indicated that patients with low immune signature score might be suitable for ICI treatment. Besides, we compared the expression levels of immune checkpoints and their ligands between the high-risk score group and low-risk score group (Fig. 8f–h). Patients with low-risk scores had modestly increased PD-L1 (mean expression level: 1.94 in the low-risk score group and 1.88 in the high-risk score group) and significantly elevated PD-1 (*p* = 0.002) and CTLA-4 (*p* < 0.0001).

## Discussion

In the present study, we constructed a prognostic immune signature using TCGA LUAD dataset. This signature consisted of 17 IRGs. Among the 17 genes, it has been reported that *BIRC5*, *S100P*, and *ADRB2* could preciously predict the outcomes of NSCLC patients [24–26]. For other IRGs, such as *LGR4*, *GDF10*, *GREM1*, *IL20RB*, *INHA*, *VIPR1*, and *ADM2*, they had been verified to participate in carcinogenesis and affect patients’ prognoses in other cancers, although the relevant studies were rare in lung cancer [27–34]. Notably, most selected IRGs could regulate cancer initiation and development by simultaneously modulating the status of the tumor immune microenvironment and the malignant biological properties of tumor cells [35]. For example, LGR4 (encoded by *LGR4*) and its ligands R-spo1-4 are not only a vital axis for tumor growth and metastasis but also promotes macrophage M2 polarization and tumor-associated macrophage (TAM) formation [35]. Besides, *ADRB2-*encoded protein β2-adrenergic receptor (β2-AR) are widely expressed on activated and memory CD8^+^ T cells [36]. β2-AR could mediate cancer immunosuppression by reprogramming the metabolism of activated T cells [36, 37]. Additionally, as an innate pattern recognition receptor, Toll-like receptor 8 (TLR8) (encoded by *TLR8*) could enhance cytokines secretion and promote anti-tumor immunity [38]. Activated TLR8 pathway remarkably reshapes the tumor immune microenvironment by decreasing infiltrating myeloid-derived suppressor cells (MDSCs), regulatory T cells (Tregs), and immunosuppressive markers, such as CTLA-4 [38]. Meanwhile, stimulating TLR8 signal could increase the numbers of tumor-infiltrating M1 monocytes and T cells [38].

Generally, previous predictive models for prognostic stratification focused on the intrinsic features of tumors, such as tumor size, lymph node metastasis, and distant metastasis. Actually, some elements of innate immunity and adaptive immunity actively participate in cancer development as well [39]. Effectors including cytotoxic T cells, B cells, and NK cells destroy tumor cells while Tregs, MDSCs, and TAMs can orchestrate immune escape and tumor growth [40]. A study in human colon cancer showed that the immunologic parameters (tumor-infiltrating immune cells’ types, locations, and numbers) could more effectively predict patients’ survival than traditional histopathological methods [41]. ‘Immunoscore’ is a quantitative forecasting tool based on tumor immune contexture which is under clinical studies in multiple cancer types as a supplement for the current histopathological staging system [42, 43]. Similar to ‘Immunoscore,’ our immune signature could also reflect the tumor immune microenvironment status and herald patients’ survival based on RNA-sequencing data. Apart from patients’ survival, this immune signature was also a predictor for patients’ response to ICI treatment. Because the information on ICI treatment was not available in TCGA LUAD dataset, we used IPS as a surrogate for ICI treatment efficacy. IPS was developed mainly based on TCGA RNA-seq profiles, which can quantitatively predict patients’ response to anti-PD-1/PD-L1 and anti-CTLA-4 therapies [23]. The IPS values were significantly higher in the low-risk score group, which indicated that this 17-IRG signature might be useful for patient selection before ICI treatment. Up till now, PD-L1 expression, TMB, mismatch repair deficiency, microsatellite instability had been applied to select patients prior to ICI therapy [8]. In the present study, the predictive capability of this immune signature is independent of TMB. On the contrary, we found that the TMB was markedly lower in the low-risk score group. Considering that IPS is a complicated model containing multiple factors, we supposed that other variables, such as upregulated immune checkpoint signals, might contribute to the enhanced treatment effect in the low-risk score group.

According to the cancer-immunity cycle theory, anti-cancer immune response consists of multiple stepwise processes, including releasing antigens of cancer cells, captured and processed by antigen presentation cells, activation of T cells, trafficking and infiltrating of T cells into tumor, recognizing and killing cancer cells [44]. During cancer development, one or more steps are hampered, such as increased expressions of immune checkpoints and their ligands, impaired T cell infiltration, as well as antigenic modulation [44, 45]. Therefore, in the condition that immune checkpoint is not the only rate-limiting step, patients might get little benefit from ICI treatment. In the present study, patients with low-risk scores had higher expressions of immune checkpoint molecules. Increased levels of immune checkpoints, such as PD-1 and CTLA-4, indirectly indicated the preexisted T cell activation for the low-risk score group. Thus, patients with low-risk scores might be more sensitive to ICI treatment.

In spite of some positive results, some questions still remained. First, this immune signature was constructed based on public datasets. The predictive capability needs further verification in randomized controlled trials. Besides, we used the IPS value to mimic patients’ response to ICI treatment. Although the correlation between IPS and response to ICI therapy had been validated in several independent datasets, IPS values still could not completely replace real treatment response.

## Conclusion

We constructed a 17-IRG prognostic model to predict LUAD patients’ survival and response to ICI treatment. Patients with low-risk scores had better prognosis and may be predicted to benefit with ICI therapy. This immune signature might be valuable for prognostic stratification and patient selection before ICI treatment. We believe this predictive model should be prospectively validated.

## Data Availability

TCGA LUAD gene expression profiles and patients’ clinical data in this study are available at UCSC Xena (https://xena.ucsc.edu/). Gene mutation data could be acquired from TCGA data portal (https://portal.gdc.cancer.gov/). The patients’ IPS values are available in the Cancer Immunome Atlas (https://tcia.at/home).

## Abbreviations

ALK: Anaplastic lymphoma kinase
β2-AR: β2-Adrenergic receptor
CTLA-4: Cytotoxic T-lymphocyte-associated antigen 4
EGFR: Epidermal growth factor receptor
GO: Gene ontology
KEGG: Kyoto Encyclopedia of Genes and Genomes
ICI: Immune checkpoint inhibitor
IRG: Immune-related gene
LASSO: Least absolute shrinkage and selection operator
LUAD: Lung adenocarcinoma
MDSC: Myeloid-derived suppressor cell
NSCLC: Non-small cell lung cancer
PD-1: Programmed cell death 1
PD-L1: Programmed cell death-ligand 1
TAM: Tumor-associated macrophage
TCGA: The Cancer Genome Atlas
TLR8: Toll-like receptor 8
TMB: Tumor mutation burden
Treg: Regulatory T cell

## Code availability

R software (4.0.0) could be downloaded from https://www.r-project.org/. Some packages used in the study are available at Bioconductor (www.bioconductor.org/).
